# Oligo(ethylene glycol) Methacrylate Copolymer-Modified Liposomes for Temperature-Responsive Drug Delivery System

**DOI:** 10.3390/molecules29235511

**Published:** 2024-11-21

**Authors:** Maria Isabel Martinez Espinoza, Sezen Gül, Luisa Mugnaini, Francesco Cellesi

**Affiliations:** Department of Chemistry, Materials and Chemical Engineering “G. Natta”, Politecnico di Milano, Via Mancinelli 7, 20131 Milan, Italy

**Keywords:** thermosensitive liposomes, thermo-responsive polymers, drug delivery systems, nanocarriers, LCST

## Abstract

A thermoresponsive copolymer based on oligo(ethylene glycol) methacrylate, Chol-P(MEO_2_MA-co-OEGMA), was synthesized using Atom Transfer Radical Polymerization (ATRP) and incorporated into thermosensitive liposomes (TSLs) for controlled drug release. The copolymer exhibited a lower critical solution temperature (LCST) of 37 °C, making it suitable for biomedical applications requiring precise thermal triggers. The copolymer was incorporated into various TSL formulations alongside phospholipids such as DPPC, Lyso-PC, HSPC, and DSPC. Physicochemical characterization of the liposomes, including average size, polydispersity index, loading efficiency (LE), and encapsulation efficiency (EE), was performed using dynamic light scattering and fluorescence spectroscopy. The results showed that the incorporation of the copolymer slightly affected particle size and decreased LE and EE in most formulations. Lyso-PC-containing formulations exhibited lower LE and EE, likely due to instability during purification. Albumin encapsulation demonstrated lower LE compared to the smaller carboxyfluorescein drug model, highlighting the influence of molecular weight on loading. Although copolymer-modified liposomes showed reduced loading capacity, they enhanced thermoresponsiveness in HSPC-based formulations. These findings suggest that incorporating thermoresponsive polymers into TSLs can optimize drug delivery systems for targeted, thermally triggered release.

## 1. Introduction

Among the various nanocarrier-based drug delivery systems, liposomes have led the transition from theoretical concepts to clinical applications [1,2,3,4,5]. Liposomes have been the subject of extensive research for many years due to their numerous advantages. They are fully biodegradable, non-toxic, and non-immunogenic [2,3,4]; they allow for the encapsulation and controlled release of a wide range of drugs, including hydrophilic, hydrophobic, and amphiphilic compounds [6,7]; they enhance drug bioavailability while reducing toxic side effects on healthy cells [5,8,9]; they improve both the pharmacokinetics and pharmacodynamics of therapeutics [10,11]. Their surfaces can be functionalized with biomolecules to improve performance, such as extended circulation times, stimuli responsiveness, and active targeting [12,13,14]. Moreover, they preferentially accumulate in tumor cells over healthy cells when their average diameter is below 200 nm, exploiting the enhanced permeation and retention (EPR) effect [15,16].

However, liposomal drug delivery systems also face several challenges that must be addressed to enhance their therapeutic efficacy. These include rapid opsonization and clearance by the reticuloendothelial system (RES) upon intravenous administration, potential drug leakage at physiological temperatures due to inherent instability [16], and generally low drug encapsulation efficiencies [8,15].

Thermosensitive liposomes (TSLs), which release their encapsulated content in response to mild hyperthermia, hold significant potential to enhance the therapeutic efficacy of liposomal drug delivery systems [8,9]. This approach enables controlled and localized drug release by leveraging the initial passive accumulation of TSLs in the targeted tissues, followed by the release of the therapeutic payload upon the application of localized hyperthermia (typically around 42 °C) [8,9,17,18].

TSLs are typically composed of lipids that undergo phase transitions in response to temperatures slightly above physiological levels. Common lipid formulations include dipalmitoylphosphatidylcholine (DPPC), distearoylphosphatidylcholine (DSPC), and cholesterol, often stabilized with polymers such as DSPE-PEG2000 to enhance circulation stability [8,9,18]. These liposomes can encapsulate a variety of therapeutic agents, including small molecules, proteins, and imaging agents [8,9,19]. Upon heating, the lipid bilayer becomes permeable, allowing the controlled release of the encapsulated cargo directly at the target site, thereby minimizing systemic toxicity and improving therapeutic outcomes [18].

Despite their advantages, TSLs face several challenges [20]. Achieving the precise and uniform temperature required for activation can be difficult within the human body, leading to incomplete drug release. Additionally, the stability of TSLs in circulation is a concern, as prolonged exposure to blood components can result in premature leakage of the drug. Furthermore, optimizing the liposomal composition to balance stability with efficient release under hyperthermic conditions remains a significant challenge [18,20]. Addressing these limitations is crucial to improving the clinical applicability of TSLs in drug delivery systems.

Recent efforts have focused on introducing temperature sensitivity to liposomes through the incorporation of thermosensitive polymers exhibiting a lower critical solution temperature (LCST) [18,21]. Unlike conventional liposomes, which rely on lipid phase transitions for temperature responsiveness, these polymer-modified liposomes achieve thermosensitivity via polymer-liposome membrane interactions. Below their LCST, these polymers are highly hydrophilic, but above this threshold, they become hydrophobic, destabilizing the phospholipid bilayer and facilitating drug release. This approach addresses several limitations of traditional liposome design, including issues related to particle size, lipid composition, drug release efficiency, and the required temperature range. Additionally, polymer-modified liposomes offer a novel mechanism for targeted drug delivery by modulating liposome-cell surface interactions in response to temperature changes.

Among thermosensitive polymers, poly(*N*-isopropylacrylamide) [poly(NIPAM)] and its copolymers are the most extensively studied [18,21]. With an LCST around 32 °C, the transition temperature of poly(NIPAM) can be finely tuned by copolymerization with comonomers of varying hydrophilicity or hydrophobicity, allowing precise control over the temperature at which drug release occurs. Hydrophobic side chains or terminal groups are commonly introduced to anchor the polymer to the liposome membrane and enhance membrane interaction [21,22].

Despite the potential advantages of these polymer-modified liposomes, several challenges remain. Broad molecular weight distributions can diminish the sharpness of the thermoresponsive behavior, affecting the precision of drug release. Moreover, the polymer-to-lipid mass ratio required for effective thermoresponsiveness is often too high, which can increase the immunogenicity and toxicity of the nanocarrier in vivo [23]. Addressing these challenges is essential for advancing the clinical application of thermosensitive polymer-liposome systems.

The primary objective of this study was to evaluate and potentially optimize the loading and encapsulation efficiencies, phase transition temperatures, and release characteristics of thermosensitive liposomes, engineered with a rational design of the lipid bilayer composition. To achieve this, a range of thermosensitive liposomal formulations incorporating various lipid types were prepared, both with and without the incorporation of a thermo-responsive copolymer into their structure.

**Figure 1:**
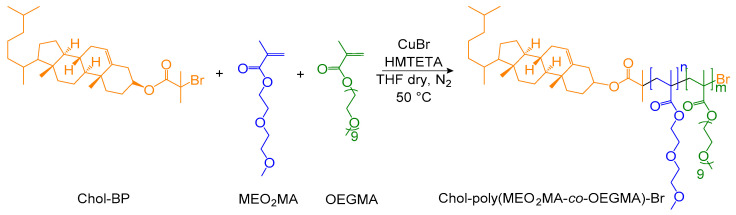
Synthesis of thermosensitive copolymer Chol-P(MEO_2_MA-co-OEGMA) obtained through ATRP.

The thermosensitive copolymer utilized in this work, Chol-P(MEO_2_MA-co-OEGMA), was synthesized via atom transfer radical polymerization (ATRP) of 2-(2-methoxyethoxy)ethyl methacrylate (MEO_2_MA) and oligo(ethylene glycol)methacrylate (OEGMA, Mw = 500 g/mol), with an initial molar ratio of [OEGMA]_0_/[MEO_2_MA]_0_ at 10:90 and an average degree of polymerization of 100, based on the method described by Lutz and Hoth [24]. In this case, we incorporated a cholesterol moiety into the copolymer structure by employing a cholesterol-functionalized initiator (Chol-BP) to facilitate attachment of the thermosensitive copolymer to the lipid bilayer of the liposomes (Figure 1).

The various liposomal formulations used in this work were designed and characterized in accordance with the recently published nanomedicine framework for successful clinical translation [25]. Firstly, a variety of thermosensitive liposomal (TSL) formulations were synthesized using the thin-film hydration method followed by extrusion. These formulations, featuring diverse lipid compositions—with or without the thermo-responsive copolymer—were designed to encapsulate the fluorescent dye carboxyfluorescein (CF) and FITC-conjugated Bovine Serum Albumin (FITC-BSA). The formulations were then evaluated for their loading and encapsulation efficiencies and release characteristics. The lipids employed in various combinations included DPPC, DSPC, lysophosphatidylcholine (Lyso-PC), DSPE-PEG2000, hydrogenated soybean phosphatidylcholine (HSPC), and cholesterol. Following the analysis of CF encapsulation and release, the formulations demonstrating optimal performance were selected for further investigation. The average particle size and polydispersity index (PDI) of the liposomes were determined using dynamic light scattering (DLS), while fluorescence assays were employed to assess loading and encapsulation efficiencies, phase transition temperatures, and release profiles.

## 2. Results and Discussion

### 2.1. Synthesis and Characterization of the Thermoresponsive Copolymer

The synthesis of Chol-P(MEO_2_MA-co-OEGMA) was successfully achieved with a high degree of control over the molecular weight distribution via ATRP. The cholesterol-based ATRP initiator (Chol-BP) was first obtained by esterification of cholesterol with α-bromoisobutyryl bromide [22] (Supporting Information, Figure S1). Afterwards, the random copolymerization of MEO_2_MA and OEGMA monomers, in a molar ratio [OEGMA]_0_/[MEO_2_MA]_0_ of 10:90 and a target degree of polymerization DP = 100, was achieved in THF in the presence of Cu(I)Br/HMTETA catalyst [26,27]. The success of the synthesis of the polymers was confirmed by ^1^H-NMR and SEC.

**Figure 2:**
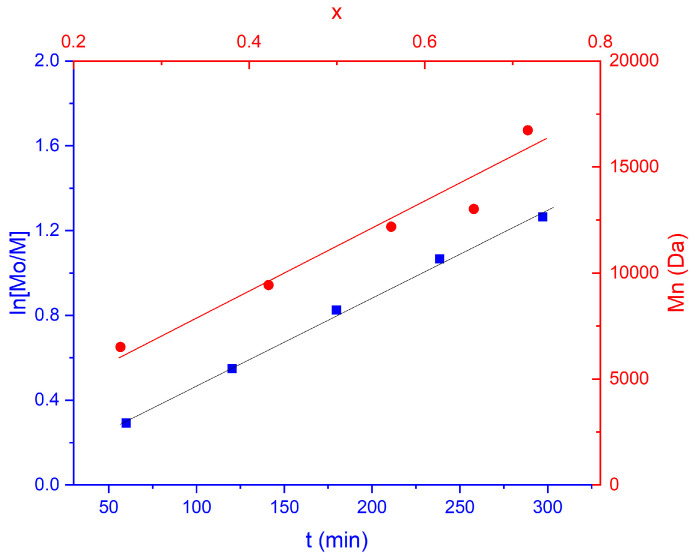
Kinetic plot of ln([M0]/[M]) versus time for ATRP of Chol-P(MEO_2_MA-co-OEGMA) (**left**); number average molecular weight (Mn) vs. conversion (X) (**right**).

The control of the polymerization, monitored via ^1^H-NMR, was confirmed by a semilogarithmic kinetic plot, which presented a linear trend typical of the first-order kinetics, and by the linear growth of the number average molecular weight with the monomer conversion (Figure 2). The ^1^H-NMR spectrum of the purified Chol-(MEO_2_MA-co-OEGMA) revealed the successful copolymerization of the monomers (Figure S2, Supporting Information), which presents the typical -CH_3_ protons of the main backbone (*δ* = 1.09–0.77 ppm) and of the termini of both MEO_2_MA and OEGMA units (3.36 ppm), and the CH_2_ protons of the ethylene oxide units of both monomers (3.57 ppm). The final Mn calculated was 17,200 g/mol (in agreement with a monomer conversion of 80%) and a dispersity Ð = 1.21 (calculated by SEC), in agreement with the ATRP conditions.

**Figure 3:**
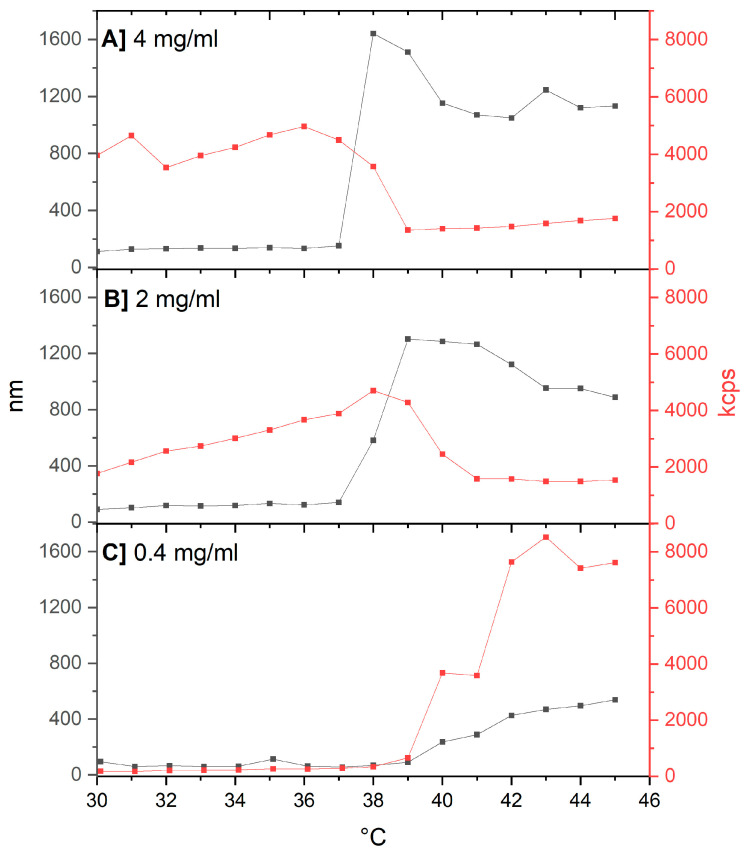
LCST analysis of Chol-P(MEO_2_MA-co-OEGMA) water solutions by DLS: Z-Average size (**left**) and average scattering intensity (**right**) vs. temperature for copolymer concentrations of 4 mg/mL (**A**), 2 mg/mL (**B**), and 0.4 mg/mL (**C**).

The LCST of the thermoresponsive copolymer was evaluated at various concentrations in aqueous solutions. DLS data were analyzed by plotting the Z-average hydrodynamic diameter and the average scattering intensity (derived count rate) as a function of temperature in the range of 30–45 °C (Figure 3). As expected, the LCST exhibited concentration dependence [28]. At a concentration of 0.4 mg/mL, the phase transition initiated at approximately 39 °C, with both the Z-average diameter and the scattering intensity increasing steadily from this transition point up to 45 °C. At higher concentrations (2–4 mg/mL), the onset of the phase transition occurred at a lower temperature (~37 °C). Although the Z-average diameter continued to increase above this temperature, the scattering intensity showed a tendency to decrease, likely due to particle sedimentation at elevated concentrations.

In this study, the concentration of the thermosensitive copolymer used to prepare polymer-modified TSL formulations was maintained above 2 mg/mL to ensure a sharp thermal transition at 37 °C, which is optimal for biomedical applications. This temperature is lower than the typical transition temperatures of the thermosensitive lipid formulations tested (>38 °C), allowing for a clearer evaluation of the copolymer’s impact on these thermal transitions.

### 2.2. Liposomal Formulations

Liposome suspensions from various lipid formulations were prepared using the lipid film hydration followed by the extrusion method. The nomenclature and composition of these formulations are summarized in Table 1.

In this study, the primary component used in all formulations was DPPC phospholipid, which has a phase transition temperature (Tm) of 41.4 °C [8]. This Tm facilitates triggered drug release under mild hyperthermia, minimizing thermal damage to surrounding tissues. However, pure DPPC liposomes are known to exhibit limited and slow drug release rates [17,29,30]. Therefore, additional phospholipids, such as Lyso-PC, HSPC, and DSPC, were incorporated to assess their effects on drug loading and release. Furthermore, DSPE-PEG2000 was added to most formulations to enhance stability and increase drug release upon heating [17,29,30,31]. Given that 4 mol% DSPE-PEG2000 is sufficient to coat the liposomal surface and reduce clearance by the reticuloendothelial system (RES) [32], we utilized 3–4 mol% in our formulations.

For the first formulation type, LTSL, Lyso-PC was incorporated into DPPC to develop thermosensitive liposomes (TSLs) capable of burst drug release. Due to its specific molecular geometry, Lyso-PC accumulates at grain boundaries near the Tm of the liposomal system, stabilizing nanopores and significantly enhancing drug release [17,30]. Additionally, Lyso-PC incorporation has been reported to lower the Tm of TSLs [33]. DSPE-PEG2000 was also added to the bilayer, as its co-presence with Lyso-PC aids in the formation and stabilization of membrane pores [34], accelerating release kinetics [35]. However, the combination of Lyso-PC and DSPE-PEG2000 may reduce TSL stability at physiological temperatures (37 °C), potentially causing premature drug leakage [32].

For the second (HTSL) and third (TTSL) formulations, HSPC (Tm = 52 °C) and DSPC (Tm = 55 °C) were respectively added to DPPC [30]. The incorporation of these higher Tm lipids can increase the liposomal Tm to 43–45 °C [36], requiring higher temperature to trigger drug release. Elevated heat transfer, however, risks the necrosis of healthy tissues surrounding the target site. Therefore, optimizing these formulations to achieve a Tm between 39–42 °C while maintaining rapid drug release is crucial [30]. To improve serum stability, cholesterol (Chol) was also included in these formulations. However, increasing the Chol content from 10% to 20% was reported to slightly broaden and decrease the Tm, as well as reduce drug release [35].

The thermoresponsive copolymer Chol-P(MEO_2_MA-co-OEGMA), with a LCST of 37 °C in ultrapure water, was also incorporated into various TSL formulations using its hydrophobic cholesterol anchor. This copolymer was supposed to enable triggered content release via its coil-to-globule phase transition near the LCST, enhancing release kinetics and improving liposomal stability.

### 2.3. Preparation and Physicochemical Characterization of CF-Loaded Liposomes

Encapsulation of the hydrophilic, low molecular weight CF was carried out to verify the loading capacity and the thermosensitive behavior of the different liposomal formulations. CF was loaded into liposomes through the thin lipid film hydration method by hydrating the dry lipid film with a CF solution (100 mM in HBS, pH 7.4) prior to the freeze-thawing and extrusion steps. Unentrapped molecules were successfully separated by gel filtration chromatography [8].

The average size, polydispersity index measured by DLS, drug loading LE%, and encapsulation efficiency EE% of CF-loaded liposome suspensions at 25 °C are summarized in Table S1 (Supporting Information).

**Figure 4:**
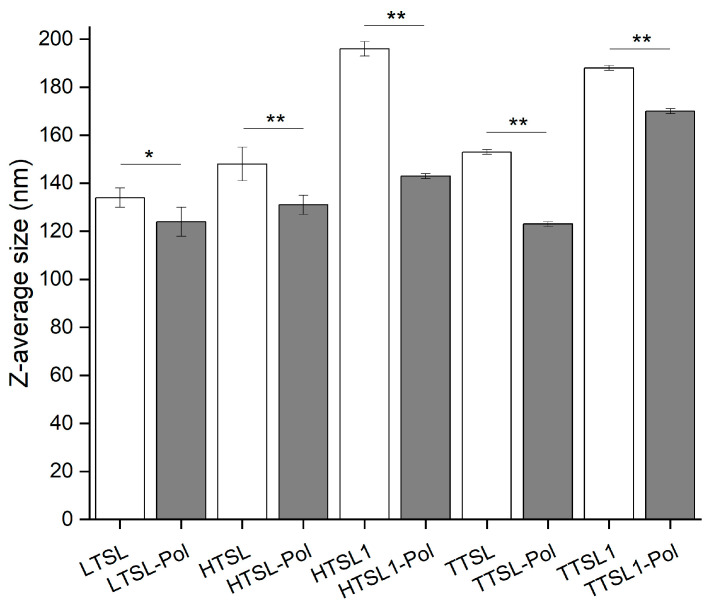
Z-average size (nm) of the different liposomal suspensions (0.5 mM) at 25 °C. (N = 3, * *p* < 0.1, ** *p* < 0.05).

The average size of the liposomes ranged from 120 to 200 nm. In general, the presence of the copolymer tended to reduce the final particle size (Figure 4). This effect may be attributed to the increased rigidity of the polymer-containing bilayer, which limits the deformation of the lipidic vesicles during extrusion through the 100 nm pores of the polycarbonate membrane.

#### 2.3.1. The Effect of Lipid Composition on LE and EE

In the case of LTSL, the maximum LE and EE were approximately 10% and 2%, respectively, after full purification and storage. For HTSL and TTSL, the maximum LE and EE values were around 20% and 4%, respectively, effectively doubling those of LTSL. This difference may be attributed to the higher instability of Lyso PC-containing liposomes, which could be significant during the purification process via size exclusion chromatography, where payload leakage may occur. Moreover, the incorporation of HSPC in place of DSPC in the formulation (HTSL vs. TTSL) did not result in significantly higher or lower LE or EE values.

When DSPE-PEG2000 was removed from the HTSL and TTSL formulations (HTSL1 and TTSL1, respectively), a decrease in LE and EE was observed in both cases, suggesting that DSPE-PEG2000 plays a key role in stabilizing the lipid bilayer and preventing payload leakage during purification.

#### 2.3.2. The Effect of Thermosensitive Copolymer on LE and EE

**Figure 5:**
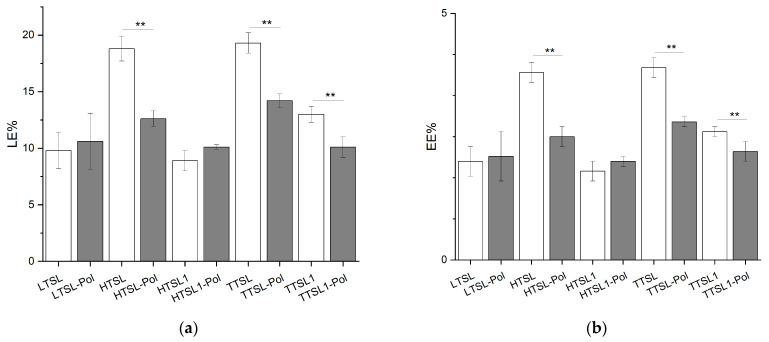
(**a**) Loading efficiency (LE%) and (**b**) encapsulation efficiency (EE%) of the different liposomal formulations. (N = 3, ** *p* < 0.05).

The impact of the thermoresponsive copolymer on the loading and encapsulation efficiency of CF-loaded liposomes was evaluated by testing various liposomal formulations (Figure 5). For the LTSL formulation, no significant difference was observed between the copolymer-modified (LTSL-Pol) and unmodified (LTSL) versions, with both exhibiting similar LE (~10%) and EE (~2%) values.

In contrast, the HTSL formulation showed a notable decrease in LE and EE upon copolymer incorporation, with a 33% reduction. Similarly, the TTSL formulation demonstrated a 26% reduction in LE and EE with the addition of copolymer. Interestingly, the copolymer-modified HTSL-Pol and TTSL-Pol formulations exhibited comparable LE (13–14%) and EE (2.5–3%) values.

When DSPE-PEG2000 was excluded, the presence of the copolymer resulted in only a minor decrease in LE and EE values for both HTSL1 and TTSL1 formulations, suggesting a reduced impact of the copolymer in the absence of this stabilizing PEGylated lipid.

The incorporation of the copolymer may have complex effects on various physicochemical properties of liposomal vesicles, including membrane thickness, rigidity during extrusion, stability during purification, interactions with the payload, particle size, and core volume. These alterations may significantly influence the loading capacity of the nanoformulations. Additionally, the association of the copolymer with the lipid membrane may vary depending on the liposomal formulation, resulting in either efficient or inefficient incorporation into the lipid bilayer.

Our findings indicate that the most pronounced impact of the copolymer on encapsulation was observed in formulations with the highest LE and EE. Notably, liposomal formulations that exhibited greater stability during extrusion and purification, and thus higher loading capacity (HTSL and TTSL), were adversely affected by copolymer incorporation.

When the CF concentration of the hydration solution was decreased below 50 mM for all of the tested formulations, the loading efficiency was significantly decreased (at least halved), whereas the encapsulation efficiency either did not exhibit a considerable change or slightly decreased (Supporting Information, Table S2).

In this case, the LE and EE values were generally too low to enable further investigation of the thermosensitive copolymer’s effect on payload release. Therefore, temperature-dependent CF release tests were conducted using formulations hydrated with a 100 mM CF solution to ensure adequate encapsulation.

### 2.4. FITC-BSA Encapsulation

To further investigate the LE and EE of higher molecular weight payloads, FITC-BSA was encapsulated into LTSL and HTSL liposomes—two formulations that exhibited distinct behaviors in terms of drug loading. BSA, which is used as a model protein in many different studies, is a water-soluble protein with a molecular weight of nearly 66 kDa [37] and a hydrodynamic diameter of approximately 7 nm [38]. FITC-tagged version of BSA was employed to take advantage of the easy protein quantification by fluorescence [39].

The particle size of FITC-BSA-loaded liposomes was narrowly distributed in the range of 100–145 nm, depending on the formulation and on the absence or presence of the copolymer (Table 2). Similar to the observations with CF encapsulation, the average size of copolymer-modified liposomes was smaller than that of the unmodified liposomes, with a reduction of approximately 20 nm.

In terms of LE, the values for FITC-BSA-loaded liposomes were significantly lower compared to those of CF-loaded liposomes. This difference is likely due to the much higher molecular weight of FITC-BSA (~70 kDa), which is two orders of magnitude greater than that of CF (376 g/mol). Among the formulations, the unmodified lysolipid-based LTSL showed the highest LE and encapsulation efficiency (EE), at approximately 0.06% and 41%, respectively. In contrast, the LE and EE values for the HTSL formulation were about half of those observed for LTSL, possibly due to the presence of cholesterol in the HTSL formulation. Cholesterol incorporation into the lipid bilayer is known to increase membrane rigidity and reduce the internal aqueous volume, which can negatively affect loading [40]. Additionally, a small portion of BSA was reported to associate tightly with the lipid membrane, similar to a transmembrane protein, while the remaining albumin molecules were encapsulated within the aqueous core of the liposomes [41,42,43]. Based on the literature, it can be inferred that cholesterol may limit the association of BSA with the lipid membrane.

When comparing the copolymer-modified formulations to their original counterparts, similar LE and EE values were observed in the HSPC-containing formulations (HTSL). In contrast, the lysolipid-based formulations LTSL exhibited a significant reduction in both LE and EE following the incorporation of the copolymer. This decrease in LE and EE for LTSLs may be attributed to the copolymer’s steric hindrance and hydrophilicity, which could interfere with the hydrophobic interactions between BSA and the lipid membrane. In all instances, the amount of BSA encapsulated was insufficient for conducting further release tests, particularly given the challenges associated with detecting differences resulting from copolymer incorporation. Consequently, the temperature-dependent release tests were concentrated solely on CF-loaded liposomes.

### 2.5. Temperature-Dependent CF Release Tests and the Effect of the Thermosensitive Copolymer

**Figure 6:**
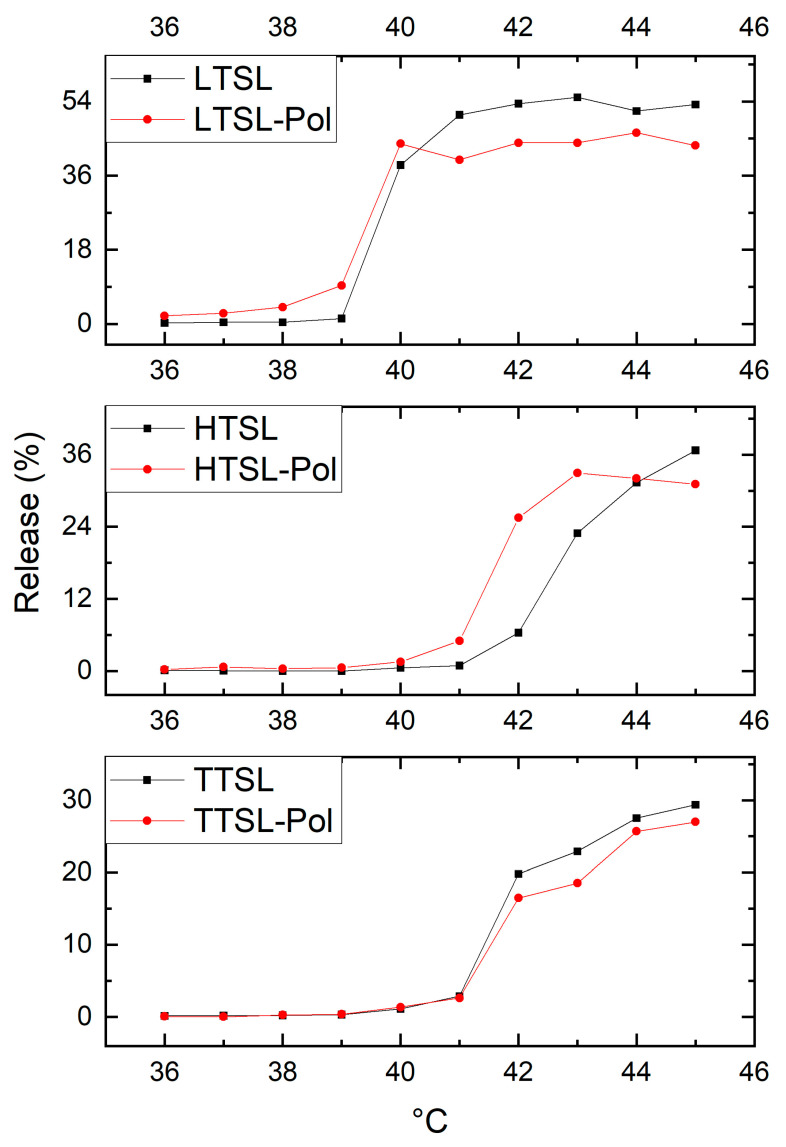
Temperature-dependent release of CF from liposomes LTSL and LTSL-Pol, HTSL and HTSL-Pol, TTSL, and TTSL-Pol. The suspensions (0.20 mM) were incubated at the desired temperature (36–45 °C) for 5 min.

A temperature dependent CF release tests (temperature range 36–45 °C) was performed to assess the phase transition temperature and the maximum release percent of the three main thermosensitive liposomal formulations (LTSL, HTSL, and TTSL), with and without the addition of the copolymer. The results are highlighted in Figure 6.

In both the original formulations and their copolymer-modified versions, the Tm of the lysolipid-based formulations LTSL (~39 °C) was considerably lower compared to the other formulations containing HSPC or DSPC (HTSL and TTSL; ~41–42 °C), as expected. The incorporation of lysolipids into the bilayer is known to decrease the Tm [33], whereas the addition of HSPC (Tm = 52 °C) or DSPC (Tm = 55 °C) [30] might raise the phase transition temperature [36].

For LTSL, a burst release was observed between 39 °C and 40 °C, followed by a stable CF release of approximately 50% up to 45 °C. In contrast, for the other TSL formulations containing HSPC or DSPC, the onset of release occurred around 41 °C, with the overall release level lower than 20–30% of that of LTSL in the temperature range of 41–45 °C. This enhanced release behavior in LTSL can be attributed to the unique molecular geometry of lysolipids, which accumulate at grain boundaries and form stabilized nanopores near the Tm of the liposomes, resulting in increased drug release rates [17,30]. Furthermore, the presence of DSPE-PEG2000 in lysolipid-containing TSLs has been shown to promote nanopore formation and stabilization, leading to rapid release kinetics [34]. Additionally, liposomes containing cholesterol have been reported to exhibit a broader phase transition and lower release rates compared to their cholesterol-free counterparts [35].

In the case of the LTSL formulation, a negligible variation in the phase transition temperature was observed with the addition of the copolymer (approximately 39 °C for both the unmodified and copolymer-modified formulations). The differences in CF release between the unmodified LTSL and LTSL-POL were also minimal, with only a slight variation above the thermal transition (55% vs. 46% at 43 °C).

In the case of HTSL, a noticeable change in the phase transition temperature was observed as a result of the copolymer incorporation. While the most significant release gradient occurred between 42–43 °C for the original HTSL formulation, HTSL-Pol exhibited a sharp release increase between 41–42 °C, with higher release levels compared to the original formulation up to 44 °C.

Therefore, in this HTSL formulation, the presence of the copolymer had a clear effect on the thermoresponsiveness of the liposomes, allowing for the modification and triggering of the payload release depending on the specific application.

In the case of the TTSL formulation, no noticeable change occurred in the phase transition temperature as a result of the inclusion of the copolymer, since both TTSL and TTSL-Pol exhibited a significant variation in CF release between 41 and 42 °C, followed by an almost steady increase at higher temperatures. The release from both TTSL and TTSL-Pol over the temperature range of 41 to 45 °C was also comparable (29% vs. 27% at 45 °C). This lack of observable change in CF release kinetics may have resulted from the ineffective incorporation of the copolymer into the lipid bilayer or inadequate membrane destabilization despite the conformational change of the copolymer.

## 3. Materials and Methods

### 3.1. Materials

Reagents and solvents were purchased from Sigma-Aldrich (Merck S.P.A., Milan, Italy). 1,2-dipalmitoyl-sn-glycero-3-phosphocholine (DPPC), 1,2-distearoyl-sn-glycero-3-phosphocholine (DSPC), 1-palmitoyl-2-hydroxy-sn-glycero-3-phosphocholine (16:0 Lyso-PC), 1,2-distearoyl-sn-glycero-3-phosphoethanolamine-*N*-[methoxy(polyethyleneglycol)-2000] (DSPE-PEG2000), L-α-phosphatidylcholine hydrogenated (Soy) (HSPC), and Cholesterol (Chol) were purchased from Avanti Polar Lipids (Alabaster, AL, USA). Deionized water was obtained from the Milli-Q^®^ water purification system (Millipore, Merck S.P.A., Milan, Italy).

### 3.2. Buffers

HBS buffer (20 mM HEPES, 150 mM NaCl, pH 7.4) was prepared as follows: 4.766 g HEPES and 8.766 g NaCl were dissolved in 500 mL of deionized water, the pH was adjusted to 7.4 with 1 M NaOH or 1 M HCl, and then the volume was completed to 1 L by adding deionized water.

Tris-HCl buffer (10 mM, 0.9% NaCl, pH 8.0) was prepared as follows: 1.211 g Tris base and 9 g NaCl were dissolved in 500 mL of deionized water, the pH was adjusted to 8 with 1 M NaOH or 1 M HCl, and then the volume was completed to 1 L by adding deionized water.

### 3.3. Equipment

^1^H NMR spectra were recorded on a Bruker (Billerica, MA, USA) Avance 400 MHz instrument at 298 K, using CDCl_3_ as solvent. Chemical shifts (δ) are reported in ppm downfield from the deuterated solvent used as an internal standard, and the coupling constants (J) are expressed in Hz.

The average size, size distribution, and polydispersity index of liposomes were determined by dynamic light scattering (DLS) using a Zetasizer Nano ZS (Malvern Instruments Ltd., Malvern, UK) equipped with a 4 mW helium/neon laser at a wavelength output of 633 nm and a backscattering angle of 173° at 25 °C.

Fluorescence analysis was performed at 25 °C by using a Jasco (Tokyo, Japan) FP-8500 equipped with a 450 W Xenon short-arc excitation source.

### 3.4. Synthesis of Cholesteryl-2-Bromoisobutyrate (Chol-Br)

First, 2.5 g of Cholesterol (6.46 mmol, 1 eq) was dissolved in 125 mL of anhydrous dichloromethane (DCM) under stirring in a nitrogen atmosphere in a two-neck round-bottom flask. Then, 1.8 mL of Triethylamine (12.9 mmol, 2 eq) was added, and the mixture was cooled to 0 °C. A solution of 2-bromoisobutyryl bromide (0.96 mL, 7.8 mmol, 1.2 eq) in 10 mL of DCM was subsequently added dropwise. The reaction mixture was allowed to gradually warm to room temperature and stirred overnight. After confirming complete esterification by thin-layer chromatography (TLC), the reaction mixture was washed twice with brine, and the organic phase was dried over sodium sulfate (Na_2_SO_4_), filtered, and concentrated under reduced pressure. The product was precipitated in ethanol, filtered, and dried under vacuum to give an 80% yield.

^1^H NMR (400 MHz, CDCl_3_), δ (ppm): δ = 0.70 (s, 3 H, cholesteryl CH_3_), 0.90 (d, 6 H, cholesteryl CH_3_), 0.95 (d, 3 H, cholesteryl CH_3_), 1.10 (s, 3 H, cholesteryl CH_3_), 0.95–2.40 (m, 28 H, cholesteryl CH and CH_2_), 1.90 (s, 6 H, CH_3_CBr), 4.60 (m, 1 H, CHO), and 5.40 (s, 1 H, CH=C).

### 3.5. Synthesis of the Thermosensitive Copolymer

THF (inhibitor free) was degassed under nitrogen for 10 min. A catalyst solution was prepared by dissolving 100 mg of copper(I) bromide (CuBr) in 2 mL of degassed THF, followed by the addition of 189 µL of 1,1,4,7,10,10-hexamethyltriethylenetetramine (HMTETA). In a Schlenk tube equipped with a magnetic stir bar, 1.27 g of 2-(2′-methoxyethoxy)ethyl methacrylate (MEO_2_MA) (6.72 mmol, 90 eq), 0.37 g of poly(ethylene glycol) methyl ether methacrylate (Mn 500) (OEGMA) (0.75 mmol, 10 eq), and 40 mg of Chol-BP initiator (0.075 mmol, 1 eq) were introduced. The monomer/Chol-BP system was degassed via four vacuum/N_2_ cycles and maintained under a nitrogen atmosphere. Degassed THF (1.56 mL) was subsequently added, followed by the addition of 0.215 mL of the catalyst solution, achieving a final molar ratio of [CuBr]/[HMTETA]/[Chol-BP] = 1/1/1. The reaction mixture was stirred at 50 °C under a nitrogen atmosphere for 6 h. After the reaction, the Schlenk tube was cooled in an ice bath, and the system was opened to air to quench the catalyst. The reaction mixture was then filtered through an alumina column using dichloromethane (DCM) as the eluent to remove residual copper salts. Finally, the solvent was removed under vacuum, and the resulting oil was precipitated in diethyl ether to yield the purified product Chol-P(MEO_2_MA-co-OEGMA) (yield = 80%), which was analyzed by ^1^H NMR and SEC.

Monomer conversions were determined by ^1^H NMR (400 MHz, CDCl_3_), comparing the integrals of the vinyl protons of the unreacted monomers (5.56 and 6.12 ppm) with the integrals of the region from 3.90 to 4.40 ppm, which corresponds to two protons from the unreacted monomers and two protons from the copolymer, under the assumption that both monomers exhibit comparable reactivity.

^1^H NMR (400 MHz, CDCl_3_) of the purified product, δ (ppm): *δ* = 4.07, bs, -CH_2_OCO; *δ* = 3.57, m, -OCH_2_CH_2_O; *δ* = 3.36, s, -OCH_3_; *δ* = 1.97–1.71, m, CH_2_ main chain; *δ* = 1.09–0.77 CH_3_ main chain. Mn_NMR_ = 17,200 g/mol, Mn_SEC_ = 24,800 g/mol, Ð = 1.21.

### 3.6. Characterization of the Lower Critical Solution Temperature (LCST)

The LCST of Chol-P(MEO_2_MA-co-OEGMA) was determined across a range of concentrations (0.4–4 mg/mL) using DLS analysis. Temperature-dependent analysis was conducted by measuring the Z-average size and scattering intensity (derived count rate) at various temperatures. The temperature was scanned from 30 °C to 45 °C in 1 °C increments, with an equilibrium time of 60 s between each measurement to ensure stability.

### 3.7. Preparation of Liposomal Suspensions

Liposomal suspensions of various lipid compositions (Table 1) were prepared using the thin-film hydration method followed by extrusion. Three primary TSL formulations were selected with the following lipid molar ratios: DPPC/Lyso-PC/DSPE-PEG2000 = 90/10/4 (LTSL), DPPC/HSPC/Chol/DSPE-PEG2000 = 50/25/15/3 (HTSL), and DPPC/DSPC/Chol/DSPE-PEG2000 = 50/25/15/3 (TTSL). Subsequently, the thermosensitive copolymer Chol-P(MEO_2_MA-co-OEGMA) was incorporated into each of these three formulations (LTSL-Pol, HTSL-Pol, and TTSL-Pol) to analyze the effect of the copolymer on payload encapsulation and thermo-responsive characteristics.

Briefly, the liposomes were prepared as follows: lipids at the desired ratios (and the copolymer for polymer-modified liposomes) were dissolved in 8 mL of a DCM/MeOH (9:1 *v*/*v*) solution. The solvent was removed by rotary evaporation at 25 °C under gradually reduced pressure, leaving a thin lipid film in a round-bottom flask. To ensure complete solvent removal, the lipid film was further dried at 30 °C under vacuum overnight in a drying oven. The dry film was then hydrated with either a 100 mM CF solution (pH 7.4 in HBS) or a 2 mg/mL FITC-BSA solution (pH 7.4 in HBS) to achieve a final lipid concentration of 20 mM. After hydration at 45 °C, the multilamellar vesicles underwent six freeze-thaw cycles (freezing in liquid nitrogen and thawing in a 45 °C water bath) to maximize the encapsulation of the payload. This was followed by 15 extrusion cycles using an Avanti^®^ mini extruder at 45 °C through a polycarbonate membrane with a pore diameter of 100 nm, yielding unilamellar vesicles with a homogeneous size distribution. Free payloads and copolymer were removed by size exclusion chromatography on a Sepharose CL-4B column (d = 1 cm, h = 15 cm) at 25 °C, using an HBS solution (20 mM HEPES, 150 mM NaCl, pH 7.4).

### 3.8. Physicochemical Characterization

Liposome suspensions were diluted to 0.5 mM and the average size and polydispersity index of the colloids were measured by DLS.

The loading efficiency (LE) and encapsulation efficiency (EE) of CF-loaded liposomes were determined by the following procedure: 20 µL of CF-loaded liposome solution with a lipid concentration of 1 mM was lysed by adding 180 µL of Triton X-100/HBS (1% *v*/*v*) solution, vortexing, and heating at 50 °C for 30 min. After the sample was cooled down to 25 °C, 800 µL of Tris-HCl buffer (10 mM, 0.9% NaCl, pH 8) was added, resulting in a 0.02 mM lipid concentration. Then, the fluorescence intensity of CF was measured by a spectrofluorometer (Jasco FP-8500) at an excitation wavelength (λex) of 495 nm and an emission wavelength (λem) of 516 nm.

The LE and EE of FITC–BSA-loaded liposomes were determined by the following procedure: 50 µL of FITC–BSA-loaded liposome solution with a lipid concentration of 2 mM was lysed by adding 150 µL of Triton X-100/HBS (1% *v*/*v*) solution, vortexing, and heating at 50 °C for 30 min. After the sample was cooled down to 25 °C, 600 µL of HBS buffer (pH 7.4) was added, resulting in a 0.125 mM lipid concentration. Then, the fluorescence intensity of FITC-BSA was measured by spectrofluorometer at an excitation wavelength of 495 nm and an emission wavelength of 520 nm. The amount of CF or FITC-BSA entrapped in liposomes was calculated by using the corresponding fluorescence calibration curves. Finally, loading efficiency (LE), and encapsulation efficiency (EE) were computed according to Equations (1) and (2), respectively.

(1) $$LE\;\left(\%\right)=\frac{Total\;\mu mol\;of\;payload\;trapped\;in\;liposomes}{Total\;\mu mol\;of\;lipids}\;\times \;100$$

(2) $$EE\;\left(\%\right)=\frac{Total\;amount\;of\;payload\;trapped\;in\;liposomes}{Total\;amount\;of\;payload\;added}\;\times \;100$$

Data were presented as mean ± standard deviation of three different replicates and analysed for statistical significance by Student’s *t*-test.

### 3.9. In Vitro Release Tests

The temperature-dependent release characteristics of CF from liposomes were analyzed using the following procedure: 700 µL of the 0.20 mM liposomal sample was placed in a water bath at a constant temperature (T = 25 °C and T = 36–45 °C) for 5 min. After release tests were completed, the samples were quickly moved into an ice bath to prevent the further release of the payload.

The percent release of CF was calculated depending on its self-quenching property, meaning that the fluorescence of CF becomes negligible when it is encapsulated by liposomes at high concentrations. When released from liposomes, it exhibits strong fluorescent signals [44]. Finally, the percent release was determined by using the following Equation (3), where I(t) is the fluorescence intensity after incubation of liposomes at a constant temperature for a certain time period, I(0) is the fluorescence baseline (samples without heating, T = 25 °C), and I(∞) is the fluorescence intensity of CF after the lysis of liposomes with Triton X-100 at 50 °C for 30 min in order to release all the encapsulated CF.

(3) $$Release\;\left(\%\right)=\frac{I\left(t\right)-I(0)}{I\left(\infty \right)-I(0)}\;\times \;100$$

## 4. Conclusions

In this study, we successfully synthesized and characterized a thermoresponsive copolymer, Chol-P(MEO_2_MA-co-OEGMA), using ATRP with controlled molecular weight distribution and narrow dispersity. The copolymer displayed a concentration-dependent LCST, with optimal thermal transition at 37 °C, making it suitable for biomedical applications. This copolymer was incorporated into various thermosensitive liposomal formulations, which were systematically evaluated for their physicochemical properties, including particle size, loading, and encapsulation efficiency.

For CF-loaded formulations, the highest LE and EE values were observed in cholesterol-containing TSLs with HSPC or DSPC, while lysolipid-based TSLs exhibited lower values, likely due to higher colloidal instability.

The incorporation of the thermoresponsive copolymer affected the loading capacity of the liposomes. Copolymer-modified liposomes showed reduced LE and EE, particularly in formulations incorporating HSPC and DSPC lipids, where the bilayer stabilization provided by the copolymer might have hindered payload encapsulation.

FITC-BSA-loaded liposomes demonstrated significantly lower LE compared to CF, attributed to the higher molecular weight of the protein. The highest loading and encapsulation efficiencies were achieved in unmodified lysolipid-based formulations. Cholesterol’s presence in the lipid bilayer likely limited the encapsulation of FITC-BSA due to reduced membrane flexibility and aqueous core volume.

The temperature-dependent release kinetics of CF from TSLs revealed a notable decrease of approximately 1 °C in the phase transition temperature of the HTSL formulation due to the incorporation of the copolymer. Consequently, the presence of the copolymer significantly enhanced the thermoresponsiveness of the HTSL liposomes, enabling the modification and triggering of payload release tailored to specific applications. In contrast, the lack of a significant change in CF release kinetics observed in other TSL formulations may result from ineffective incorporation of the copolymer into the lipid bilayer or insufficient membrane destabilization, despite the copolymer’s conformational changes. Further optimization of copolymer concentration, lipid composition, the method of liposome preparation, and copolymer inclusion may enhance the efficacy of these TSLs for more specific drug loading and targeted delivery applications.

## Figures and Tables

**Table 1:** Nomenclature and composition of the liposomal formulations investigated.

Name	Formulation Composition	Ratio mol/mol
LTSL	DPPC: Lyso PC: DSPE-PEG2000	90: 10: 4
LTSL-Pol	DPPC: Lyso PC: DSPE-PEG2000: Copolymer	90: 10: 4: 0.7
HTSL	DPPC: HSPC: Chol: DSPE-PEG2000	50: 25: 15: 3
HTSL-Pol	DPPC: HSPC: Chol: DSPE-PEG2000: Copolymer	50: 25: 15: 3: 0.7
HTSL1	DPPC: HSPC: Chol	50: 25: 15
HTSL1-Pol	DPPC: HSPC: Chol: Copolymer	50: 25: 15: 0.7
TTSL	DPPC: DSPC: Chol: DSPE-PEG-2000	50: 25: 15: 3
TTSL-Pol	DPPC: DSPC: Chol: DSPE-PEG-2000: Copolymer	50: 25: 15: 3: 0.7
TTSL1	DPPC: DSPC: Chol	50: 25: 15
TTSL1-Pol	DPPC: DSPC: Chol: Copolymer	50: 25: 15: 0.7

**Table 2:** Average size, PDI, loading, and encapsulation efficiency of FITC-BSA-loaded liposomes (0.5 mM). Data resented as Mean ± SD, N = 3.

Name	Av. Size (nm)	PDI	LE (%)	EE (%)
LTSL	123 ± 1	0.2 ± 0.1	0.06 ± 0.01	41.0 ± 0.1
LTSL-Pol	104 ± 1	0.2 ± 0.1	0.03 ± 0.01	23.4 ± 0.5
HTSL	141 ± 1	0.1 ± 0.1	0.03 ± 0.01	18.6 ± 0.5
HTSL-Pol	122 ± 1	0.2 ± 0.1	0.02 ± 0.01	17.3 ± 3.5

## Data Availability

Data are contained within the article and Supplementary Materials.

## Supplementary Materials

The following supporting information can be downloaded at: https://www.mdpi.com/article/10.3390/molecules29235511/s1, Figure S1: ^1^H NMR spectra (400 MHz, CDCl_3_) of (A) cholesterol and (B) Cholesteryl-2-bromoisobutyrate (Chol-Br); Figure S2: ^1^H NMR spectra (400 MHz, CDCl_3_) of Chol-P(MEO_2_MA-co-OEGMA) copolymer; Table S1: Physicochemical properties of CF-loaded liposome suspensions (0.5 mM, CF 100 mM) at 25 °C. Data presented as Mean ± SD of experiments run in triplicate; Table S2: Physicochemical properties of CF-loaded liposome suspensions (0.5 mM, CF 45 mM) at 25 °C. Data presented as Mean ± SD.
