# Supplementary material for: Oligo(ethylene glycol) Methacrylate Copolymer-Modified Liposomes for Temperature-Responsive Drug Delivery System
Source: Molecules. 2024 Nov 21;29(23):5511. doi: 10.3390/molecules29235511 (PMC11643387; doi:10.3390/molecules29235511)
Supplement: Supplementary file 1 [file molecules-29-05511-s001.zip › molecules-3286081-supplementary.pdf]

Supporting Information

Oligo(ethylene glycol) Methacrylate Copolymer-Modified Liposomes for Temperature-Responsive Drug Delivery System

Maria Isabel Martinez Espinoza, Sezen Gul, Luisa Mugnaini, Francesco Cellesi*

Dipartimento di Chimica, Materiali ed Ingegneria Chimica "G. Natta". Politecnico di Milano, Via Mancinelli 7, 20131 Milan, Italy

*Corresponding author.

Email address: francesco.cellesi@polimi.it (F. Cellesi).

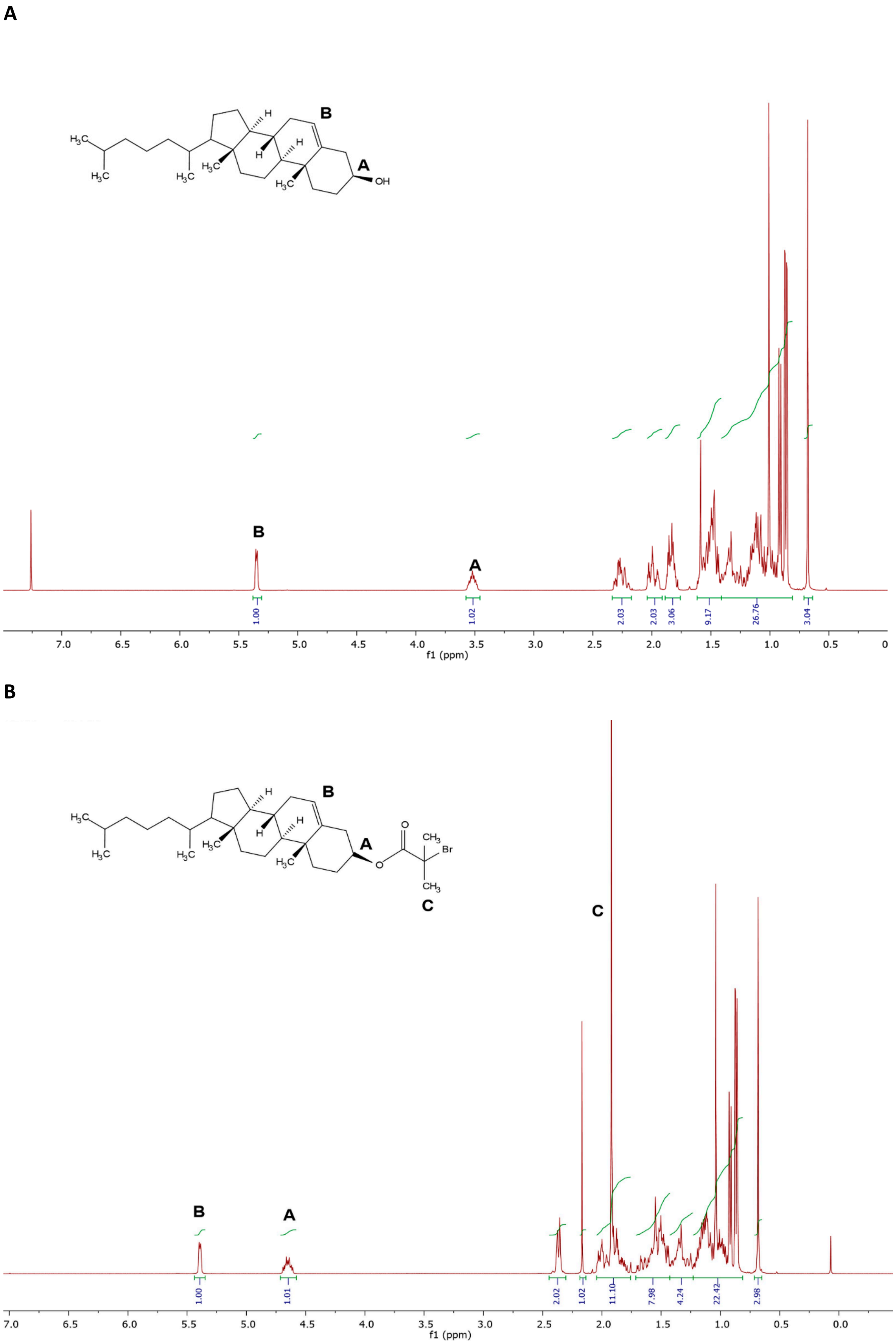

Figure S1. ^{1}H NMR spectra (400 MHz, $CDCl_3$) of (A) cholesterol and (B) Cholesteryl-2-bromoisobutyrate (Chol-Br).

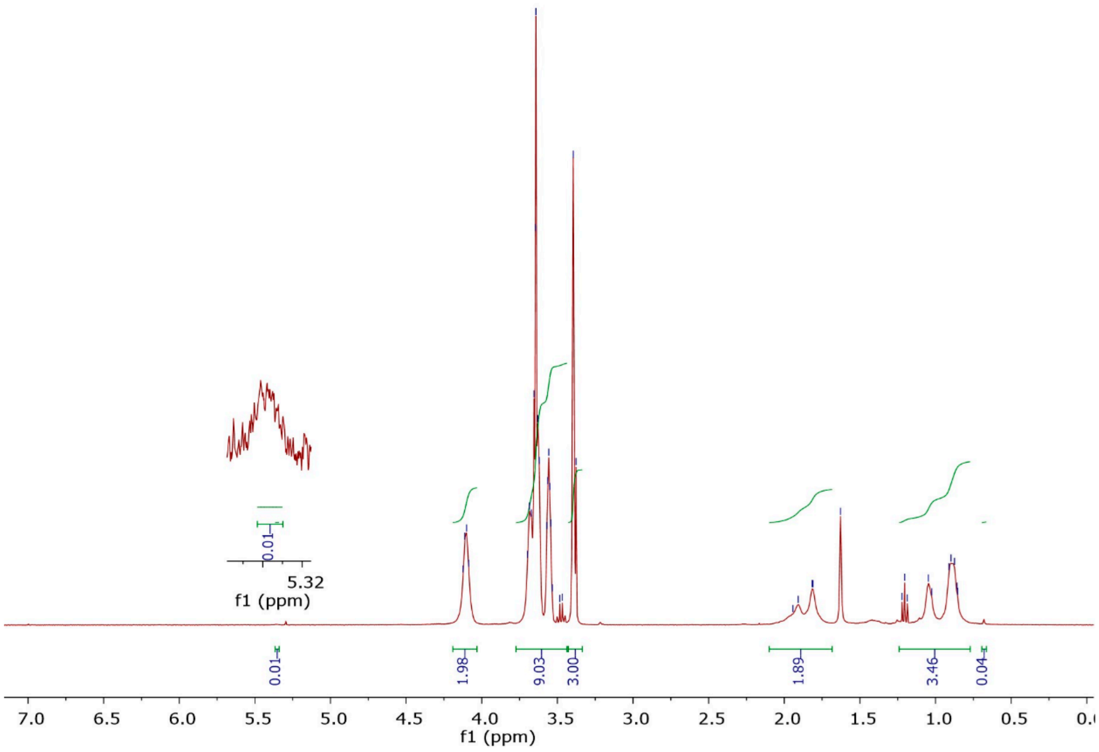

Figure S2. ^{1}H NMR spectra (400 MHz, $CDCl_3$) of Chol-P(MEO_2MA-co-OEGMA) copolymer

Table S1. Physicochemical properties of CF-loaded liposome suspensions (0.5 mM, CF 100 mM) at 25°C. Data presented as Mean ± SD of experiments run in triplicate.

Name	Composition (mol/mol)	Av. size (nm)	PDI	LE (%)	EE (%)
LTSL	DPPC: Lyso PC: DSPE-PEG2000 (90: 10: 4)	134 ± 4	0.1 ± 0.1	9.8 ± 1.6	2.0 ± 0.3
LTSL-Pol	DPPC: Lyso PC: DSPE-PEG2000: Copolymer (90: 10: 4: 0.7)	124 ± 6	0.1 ± 0.1	10.6 ± 2.5	2.1 ± 0.5
HTSL	DPPC: HSPC: Chol: DSPE-PEG2000 (50: 25: 15: 3)	148 ± 7	0.1 ± 0.1	18.8 ± 1.1	3.8 ± 0.2
HTSL-Pol	DPPC: HSPC: Chol: DSPE-PEG2000: Copolymer (50: 25: 15: 3: 0.7)	131 ± 4	0.1 ± 0.1	12.6 ± 0.7	2.5 ± 0.2
HTSL1	DPPC: HSPC: Chol (50: 25: 15)	196 ± 3	0.2 ± 0.1	8.9 ± 0.9	1.8 ± 0.2
HTSL1-Pol	DPPC: HSPC: Chol: Copolymer (50: 25: 15: 0.7)	143 ± 1	0.1 ± 0.1	10.1 ± 0.2	2.0 ± 0.1
TTSL	DPPC: DSPC: Chol: DSPE-PEG-2000 (50: 25: 15: 3)	153 ± 1	0.1 ± 0.1	19.3 ± 0.9	3.9 ± 0.2
TTSL-Pol	DPPC: DSPC: Chol: DSPE-PEG-2000: Copolymer (50: 25: 15: 3: 0.7)	123 ± 1	0.1 ± 0.1	14.2 ± 0.6	2.8 ± 0.1
TTSL1	DPPC: DSPC: Chol (50: 25: 15)	188 ± 1	0.2 ± 0.1	13.0 ± 0.7	2.6 ± 0.1
TTSL1-Pol	DPPC: DSPC: Chol: Copolymer (50: 25: 15: 0.7)	170 ± 1	0.2 ± 0.1	10.1 ± 0.9	2.2 ± 0.2

Table S2. Physicochemical properties of CF-loaded liposome suspensions (0.5 mM, CF 45 mM) at 25°C. Data presented as Mean ± SD.

Sample	Av. size (nm)	PDI (nm)	LE (%)	EE (%)
**LTSL**	153 ± 1	0.1 ± 0.1	5.3 ± 0.1	2.4 ± 0.1
**LTSL-Pol**	151 ± 1	0.2 ± 0.1	1.8 ± 0.1	0.8 ± 0.2
**HTSL**	154 ± 3	0.1 ± 0.1	5.3 ± 0.6	2.4 ± 0.3
**HTSL-Pol**	170 ± 2	0.1 ± 0.1	6.2 ± 0.1	2.7 ± 0. 1
**TTSL**	176 ± 6	0.1 ± 0.1	2.0 ± 0.1	0.9 ± 0.1
**TTSL-Pol**	180 ± 9	0.2 ± 0.1	2.3 ± 0.2	1.0 ± 0.1
